# P02.22. Acupuncture is acceptable to children and adolescents undergoing cancer therapy

**DOI:** 10.1186/1472-6882-12-S1-P78

**Published:** 2012-06-12

**Authors:** D Rooney, K Taromina

**Affiliations:** 1 Columbia University Medical Center, New York City, USA

## Purpose

Previous studies have suggested the safety of acupuncture in patients with cancer; however, the variables associated with its acceptance, feasibility, and use have yet to be described. We present an interim-analysis of a prospective study evaluating acupuncture feasibility among children and adolescents with cancer.

## Methods

Eligible participants were acupuncture-naïve children and adolescents undergoing treatment for cancer at CUMC. Upon consent, participants completed the Memorial Symptom Assessment Scale and were offered a variety of CAM. Requested therapies and reasons for acceptance and refusal were recorded every 3 weeks over a 6 month period. Participants receiving acupuncture completed questionnaires evaluating reasons for treatment, chief complaints, and perceptions of the efficacy of acupuncture before and after treatment.

## Results

Fifty-four percent of participants chose to receive acupuncture. Diagnoses of patients requesting acupuncture included: leukemia/lymphoma (22), brain tumor (6), solid tumor (4), other (4). Median age of those who received acupuncture was 14 years (range 1-23); 75% were above age 10. One hundred eighty-one sessions of acupuncture were administered. Eighty-nine percent of patients received acupuncture more than once, with a median of 3.5 sessions per patient (range 1-13). Nine percent of all sessions were in thrombocytopenic patients and nine percent were in neutropenic patients. Reasons for selecting acupuncture treatment for the first time were: “to try it”, “might work”, “curious” (40%), pain (22%), relaxation (11%), nausea (9%), and other supportive care indications (18%). Seventy-five percent of the first treatments resulted in patient-reported relief from their chief complaint. In 134 treatments, acupuncture was used simultaneously with other CAM including massage therapy.

## Conclusion

More than half of the participants accept acupuncture for varied purposes and complement its use with other CAM. The majority report beneficial effects with acupuncture and request additional sessions. These results suggest that acupuncture is well accepted among children and adolescents with cancer and should be considered for symptom management.

